# RETRACTION: MicroRNA‐498 Reduces the Proliferation and Invasion of Colorectal Cancer Cells via Targeting Bcl‐2

**DOI:** 10.1002/2211-5463.13949

**Published:** 2024-12-06

## Abstract

**RETRACTION**: T. Wang, L. Ma, W. Li, L. Ding, and H. Gao, “MicroRNA‐498 Reduces the Proliferation and Invasion of Colorectal Cancer Cells via Targeting Bcl‐2,” *FEBS Open Bio* 10, no. 1 (2020): 168‐175, https://doi.org/10.1002/2211‐5463.12767.

The above article, published online on 17 December 2019, in Wiley Online Library (wileyonlinelibrary.com), has been retracted by agreement between the journal Editor‐in‐Chief, Miguel A. De la Rosa; FEBS Press; and John Wiley and Sons Ltd. The retraction has been agreed upon following an investigation into concerns raised by a third party, which revealed inappropriate image duplications between this (Figure 3A and B, 5E and F) and other articles that were previously published or published in the same or following year. Given the extent of the identified issues, the editors have lost confidence in the data presented and consider the conclusions of this manuscript substantially compromised.

**RETRACTION**: T. Wang, L. Ma, W. Li, L. Ding, and H. Gao, “MicroRNA‐498 Reduces the Proliferation and Invasion of Colorectal Cancer Cells via Targeting Bcl‐2,” *FEBS Open Bio* 10, no. 1 (2020): 168‐175, https://doi.org/10.1002/2211‐5463.12767.

The above article, published online on 17 December 2019, in Wiley Online Library (wileyonlinelibrary.com), has been retracted by agreement between the journal Editor‐in‐Chief, Miguel A. De la Rosa; FEBS Press; and John Wiley and Sons Ltd. The retraction has been agreed upon following an investigation into concerns raised by a third party, which revealed inappropriate image duplications between this (Figure 3A and B, 5E and F) and other articles that were previously published or published in the same or following year. Given the extent of the identified issues, the editors have lost confidence in the data presented and consider the conclusions of this manuscript substantially compromised.

